# Interaction Between Bovine Serum Albumin and *Trans*-Resveratrol: Multispectroscopic Approaches and Molecular Dynamics Simulation

**DOI:** 10.3390/foods14142536

**Published:** 2025-07-20

**Authors:** Xiujuan Li, Mimi Guo, Chenxia Xie, Yalin Xue, Junhui Zhang, Dong Zhang, Zhangqun Duan

**Affiliations:** 1 Institute of Cereal & Oil Science and Technology, Academy of National Food and Strategic Reserves Administration, Beijing 100037, China; lixj@ags.ac.cn (X.L.); gmm@ags.ac.cn (M.G.); xcx@ags.ac.cn (C.X.); xyl@ags.ac.cn (Y.X.); 2 Institute of XinJiang Uygur Autonomous Region Grain and Oil Science (Grain and Oil Product Quality Supervision and Inspection Station of Xinjiang Uygur Autonomous Region), Urumqi 830000, China; 18997978290@163.com; 3 Institute of Cereal & Oil Quality and Nutrition (Institute of Feed), Academy of National Food and Strategic Reserves Administration, Beijing 100037, China

**Keywords:** bovine serum albumin, *trans*-resveratrol, multispectroscopic methods, interaction, molecular dynamics

## Abstract

Recent studies have increasingly focused on molecular interactions between small molecules and proteins, especially binding mechanisms and thermodynamics, using multispectroscopic and molecular dynamics approaches. This study elucidated the molecular interaction mechanism between bovine serum albumin (BSA) and *trans*-resveratrol (Res) through an integrated approach combining multispectroscopic analyses and molecular dynamics simulations. The fluorescence quenching study revealed a static quenching mechanism between BSA and Res, which was further confirmed via ultraviolet–visible (UV-Vis) absorption spectroscopy. In particular, K_SV_ decreased from 5.01 × 10^4^ M^−1^ at 298 K to 3.99 × 10^4^ M^−1^ at 318 K. Furthermore, the calculated K_q_ values significantly exceeded 1 × 10^12^ M^−1^ s^−1^. With increasing Res concentration, the peak fluorescence intensities of Tyr and Trp residues both exhibited a blue shift. The α-helix content of the BSA–Res complex was 59.8%, slightly lower than that of BSA (61.3%). Res was found to bind to site I in subdomain IIA of BSA. The molecular dynamics simulation also identified the specific binding of Res to site I of BSA, while thermodynamic studies revealed that the binding process occurs spontaneously and is primarily mediated by hydrogen bonding interactions. These findings not only enrich the theoretical framework of small-molecule–protein interactions but also provide a crucial scientific foundation for the development and utilization of natural products.

## 1. Introduction

**Figure 1:**
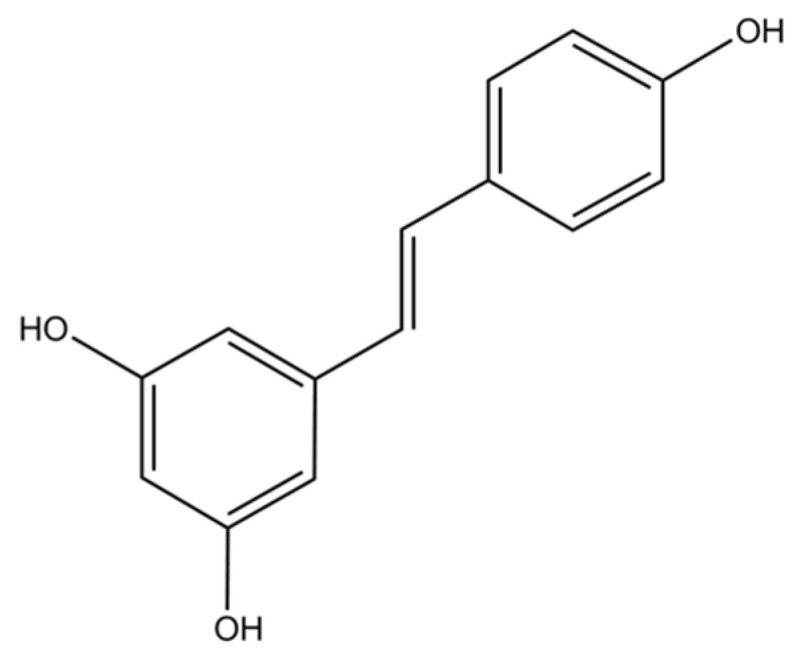
Chemical structure of *trans*-resveratrol.

*Trans*-resveratrol (3,4′,5-trihydroxystilbene, Res)—an important polyphenol compound—is characterized by two phenolic rings linked by an ethylene bridge (C=C double bond), as illustrated in Figure 1. This secondary metabolite is biosynthesized in plants as a defensive response to various biotic and abiotic stresses [1,2]. The primary botanical sources of Res include peanut, grape, Polygonum cuspidatum, blueberry, bilberry, and cranberry, among others, with grapes and peanuts representing the most abundant natural reservoirs of this compound [3]. In recent years, Res has attracted significant scientific attention due to its diverse pharmacological properties and health benefits, including antioxidant activity [4], antiviral effects [5], anti-aging properties [4,6], anti-cancer potential [7], and neuroprotective capabilities [4,8], and so on. However, despite its remarkable biological activities, the therapeutic application of Res is substantially limited by its poor bioavailability, low aqueous solubility, and chemical instability during food processing and gastrointestinal digestion [9].

Many strategies have been developed to enhance the bioavailability, solubility, and stability (thermal and pH) of bioactive compounds, such as emulsions, nanoparticles, and microencapsulations [9,10,11,12,13]. Among these strategies, protein–ligand complexes—a class of protein-based nanocarriers—have attracted considerable attention from researchers and pharmaceutical/food scientists. These complexes exhibit reversible binding characteristics due to the dissociation of the protein–ligand interaction under physiological conditions, thereby enabling controlled release of the bioactive ligand [14]. Generally, the protein and ligand interact via non-covalent bonds and hydrogen bonding [14,15]. In these systems, globular proteins have become the most extensively utilized protein carriers. One such protein is BSA, which presents superior stability and availability. Structurally, BSA comprises 583 amino acid residues, 17 disulfide bonds, and has a molecular weight of 66 kDa. BSA has three domains, each of which is divided into two subdomains. For ligand binding, three main binding sites in these subdomains (IIA, IIIA, and IB) of BSA have been successfully identified [16], and the interactions between BSA and various ligands have been extensively investigated. For instance, Hussain et al. performed comprehensive research on the binding mechanism of β-resorcylic acid with BSA, revealing that the ligand specifically bound to subdomain IIIA and induced conformational changes in BSA’s microenvironment [16]. Similar findings have been reported by Qi et al., who observed that the conformational microenvironment of BSA–lutein dipalmitate complex differed when compared to BSA alone [15]. All these studies have demonstrated the potential of BSA as an effective delivery system for various bioactive compounds. However, research on the interactions between BSA and Res remains scarce.

This study aims to elucidate the mechanisms underlying the molecular interaction between BSA and Res through an integrated approach combining multispectroscopic techniques with molecular dynamics simulation. This study facilitates deeper understanding of the formation of the BSA–Res complex and its structural dynamics, which may facilitate the development of novel delivery systems for resveratrol and other bioactive polyphenols.

## 2. Materials and Methods

### 2.1. Materials and Reagents

BSA and Res were purchased from Yuanye Bio-Tech (Shanghai, China). Warfarin, ibuprofen, and bilirubin were obtained from Merck (Shanghai, China). Chromatographic grade methanol was obtained from Fisher (Shanghai, China).

### 2.2. Sample Preparation

Approximately 200 μM of BSA was prepared in 20 mM phosphate-buffer solution (PBS). The accurate concentration of BSA was measured using an Evo 300 spectrophotometer (Thermo Fisher, Middletown, VA, USA) at 280 nm (with a coefficient of 44,720 M^−1^ cm^−1^), according to a method published elsewhere [17]. A total of 0.2282 g of Res was dissolved in ethanol solution (70/30, *v*/*v*), then diluted to 200 μM using PBS. The level of ethanol was no higher than 1% in the final system [18]. The BSA and BSA–Res complex were incubated at 298, 308, and 318 K for 10 min. After incubation, instrumental analyses were performed.

### 2.3. UV Absorption

UV absorption analysis of BSA (5 μM) and BSA–Res complex (BSA: 5 μM; Res: 5–30 μM at 5μM intervals) was performed on an Evo 300 spectrophotometer (Thermo Fisher, Middletown, VA, USA) using 10 mm quartz at 190–350 nm. The scanning speed was set as 240 nm/min.

### 2.4. Fluorescence Spectroscopy

Fluorescence spectroscopic characterization of BSA and its complex with Res was conducted using an F98 fluorescence spectrophotometer (Shanghai Lengguang Technology Co., Ltd., Shanghai, China) equipped with a 1 cm quartz cuvette. The excitation and emission slits were both set at 5 nm, and the scanning speed was set at 1000 nm/min.

Fluorescence emission spectra of BSA (5 μM) and its complexes with resveratrol (BSA: 5 μM; Res: 5–30 μM at 5 μM intervals) were acquired at three different temperatures (298, 308, and 318 K) following the experimental protocol developed by Qi et al. [15]. The spectral measurements were performed with an excitation (E_x_) wavelength of 280 nm, while recording the emission (E_m_) range from 300 to 500 nm. To account for the inner filter effect resulting from UV absorption of Res, the observed fluorescence intensities of both BSA and BSA–Res complexes were corrected using the equation (Equation (1)) established by Siddiqui et al. [19]:

(1) $$F_{adj}=F_{obs}\times e^{\frac{A_{1}+A_{2}}{2}}$$

where F_adj_ and F_obs_ denote the corrected and observed fluorescence intensities, respectively; A_1_ represents the absorbance of E_x_; and A_2_ denotes the absorbance of E_m_.

To elucidate the fluorescence quenching mechanism of Res on BSA, the Stern–Volmer equation was applied for quantitative analysis (Equation (2)) [20]:

(2) $$\frac{F_{0}}{F}=1+K_{SV}[Q]=1+K_{q}\tau_{0}[Q]$$

where F_0_ and F represent the fluorescence intensities of BSA and the BSA–Res complex, respectively. The Stern–Volmer constant (KSV) characterizes the quenching efficiency, while [Q] denotes the molar concentration of Res (M), Kq indicates the quenching rate constant, and τ_0_ indicates the mean fluorescence lifetime of biomacromolecules (10^−8^ s).

The synchronous fluorescence emission profiles of BSA (5 μM) and the BSA–Res complex (BSA: 5 μM; Res: 5–30 μM at 5μM intervals) were acquired at 298 K. Δλ was set to 15 and 60 nm for the observation of tyrosine (Tyr) and tryptophan (Trp), respectively. The excitation wavelength scanning started at 230 nm.

The three-dimensional (3D) fluorescence spectra of BSA (5 μM) and the BSA–Res complex (30 μM) were also obtained at 298 K with the excitation and emission wavelengths both ranging from 200 to 700 nm.

### 2.5. CD Spectroscopy

Circular dichroism (CD) spectroscopy analysis was performed using an MOS-450 CD spectrometer (Bio-Logic, Seyssinet-Pariset, France) with a 0.2 cm quartz cell at 190–260 nm [15]. The wavelength interval was 2 nm. PBS was used as blank. The amounts of α-helix in BSA and the BSA–Res complex were calculated using Equations (3) and (4):

(3) $$MRE=\frac{CD(\mathrm{md}\mathrm{eg})}{c\times n\times l\times 10}$$

where MRE is the mean residue ellipticity at 208 nm, c is the concentration of BSA (M), *n* is 583, and l is 0.2 (cell path).

(4) $$\alpha \text{-}\mathrm{helix}\;\mathrm{content}\;(\%)=\frac{MRE_{208}-4000}{33000-4000}\times 100$$

where MRE_208_ denotes the value of MRE at 208 nm.

### 2.6. Binding Site Determination

Site marker fluorescence probes—including warfarin, ibuprofen, and bilirubin—were used to determine the sites at which Res binds to BSA. The BSA–Res complex (BSA: 5 μM; Res: 5 μM) was treated with various concentrations of fluorescence probes at 0, 2, 4, 6, 8, 10, and 12 μM. The emissions were recorded at an excitation wavelength of 280 nm. The binding site was determined according to the literature and expressed using the F_2_/F_1_ ratio (where F_1_ and F_2_ are the emissions of the BSA–Res complex without and with the addition of probes, respectively) [21].

### 2.7. Molecular Dynamics

In this study, molecular dynamics (MD) simulation was employed to study the binding interactions between BSA and Res, focusing on their binding pattern, dynamic binding process, and amino acid energy determinants. The structure of BSA (PDB ID: 4OR0, resolution: 2.58 Å) was downloaded from the Protein Data Bank (https://www.rcsb.org/). Using the CHARMM and CGenFF force fields, BSA and Res models were constructed and optimized, respectively.

### 2.8. Statistical Analysis

All experiments and analyses were performed in duplicate. Bio-Kine 32 (Bio-Logic, Seyssinet-Pariset, France) was used for the acquisition and analysis of CD data. The SPSS software V26.0 (Softonic International, Barcelona, Spain) was used for statistical analyses. All figures were obtained using the software packages GraphPad Prism 9 (Dotmatics, CA, USA), Origin 2021 (OriginLab, Northampton, MA, USA), and Gromacs 2021 (http://www.gromacs.org).

## 3. Results and Discussion

### 3.1. UV Absorption Spectroscopy

**Figure 2:**
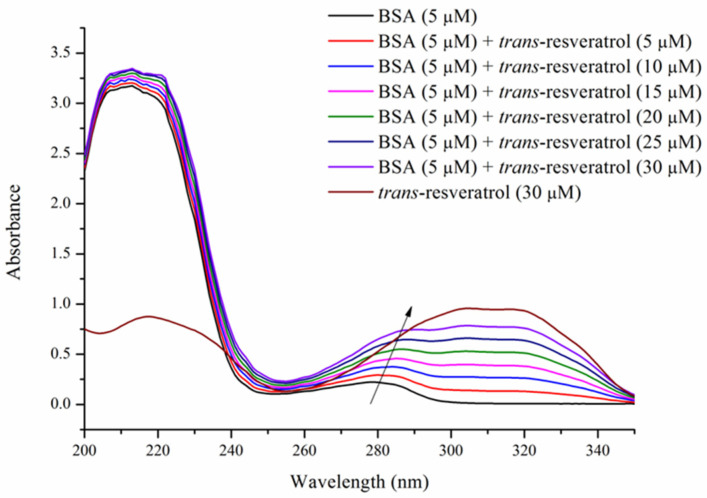
UV spectra of BSA (5 μM) treated with *trans*-resveratrol at different concentrations (0, 5, 10, 15, 20, 25, and 30 μM). T = 298 K, pH = 7.4.

BSA exhibits strong absorbance at 276–280 nm due to the π-π transitions in the benzene rings of its Tyr and Trp residues. As illustrated in Figure 2, the absorbance intensity of both BSA and the BSA–Res complex showed a significant increase accompanied by a 6 nm red shift as the Res concentration increased from 0 to 30 μM. This phenomenon indicated that Res binding induced the exposure of Tyr and Trp residues to the aqueous phase, resulting in decreased hydrophobic interactions among the hydrophobic residues of BSA. Consequently, this led to enhanced π-π conjugation and increased absorbance intensity. These results are in agreement with the observations reported by Hussain et al., who documented a similar red shift and increased UV absorbance in the BSA–β-resorcylic acid complex with increasing β-resorcylic acid concentration [14]. In contrast, Qi et al. observed a blue shift in the BSA–lutein dipalmitate complex with increasing lutein dipalmitate concentration [15]. These divergent observations may be attributed to the distinct chemical properties of the ligands and their specific interactions with BSA.

### 3.2. Fluorescence Emission

**Figure 3:**
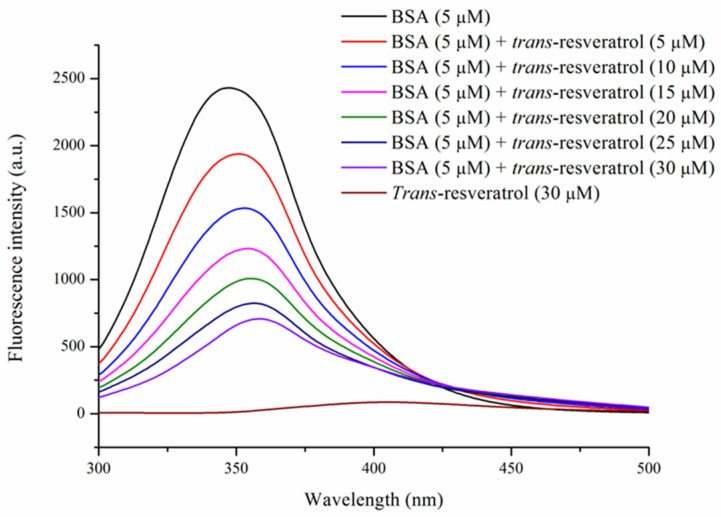
Fluorescence spectra of BSA (5 μM) treated with *trans*-resveratrol at different concentrations (0, 5, 10, 15, 20, 25, and 30 μM). T = 298 K, pH = 7.4.

Fluorescence quenching is widely employed as a sensitive technique to investigate protein–ligand interactions. The BSA–Res complex exhibited decreased fluorescence intensity and a slight red shift with increasing Res concentration (0–30 μM) (Figure 3). This phenomenon suggests that Res binding alters the microenvironment of BSA, particularly around the fluorophore residues. Kandagal et al. reported a similar fluorescence quenching effect when BSA interacted with doxepin hydrochloride [22]. Furthermore, Qi et al. and Hussain et al. observed a fluorescence intensity reduction in BSA–lutein dipalmitate and BSA–β-resorcylic acid complexes with increasing concentration of the respective ligands [14,15].

### 3.3. Fluorescence Quenching Mechanism

Fluorescence quenching mechanisms primarily include static quenching, dynamic quenching, and their combination. Static quenching involves the formation of a ground-state complex between the protein and ligand, whereas dynamic quenching results from molecular collisions in the excited state. In some cases, both mechanisms may coexist simultaneously [14]. The quenching constants (K_SV_) calculated according to the Stern–Volmer formula were used to distinguish the presence of such mechanisms. For static quenching, values decrease with rising temperature; meanwhile, for dynamic quenching, K_SV_ values increase with temperature. As presented in Table 1 and Figure S1, K_SV_ decreased from 5.01 × 10^4^ M^−1^ at 298 K to 3.99 × 10^4^ M^−1^ at 318 K. Furthermore, the calculated K_q_ values significantly exceeded 1 × 10^12^ M^−1^ s^−1^, and the maximum value of the dynamic quenching rate was constant, confirming the static quenching nature of this process. These findings are consistent with the work reported by Liang et al., who reported quenching constants in the range of 10^4^ to 10^6^ M^−1^ for the resveratrol–β-lactoglobulin complex [23].

### 3.4. BSA Conformation Analysis

#### 3.4.1. Synchronous Fluorescence Spectroscopy

**Figure 4:**
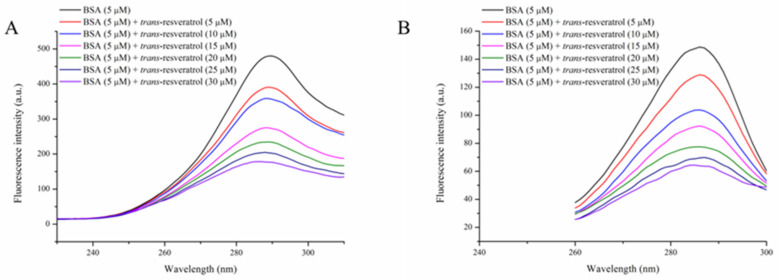
Synchronous fluorescence spectra of BSA treated with *trans*-resveratrol at different concentrations (0, 5, 10, 15, 20, 25, and 30 μM). T = 298 K, pH = 7.4. Δλ = 15 nm (**A**), Δλ = 60 nm (**B**).

The chromophores of amino acid residues of BSA are generally investigated using the synchronous fluorescence spectroscopy method, setting Δλ = 15 nm for monitoring of the Tyr residue and 60 nm for observation of the Trp residue [24]. With increasing Res concentration, the peak fluorescence intensities of Tyr and Trp residues were both slightly blue-shifted (Figure 4), indicating a decrease in polarity of the microenvironment near Tyr and Trp residues, as well as an increase in hydrophobicity. For Tyr and Trp residues, the maximum quenching percentages were 63.2% and 57.0%, respectively, indicating similar microenvironment changes near Tyr and Trp residues during the interaction process.

#### 3.4.2. Three-Dimensional Fluorescence Spectroscopy

**Figure 5:**
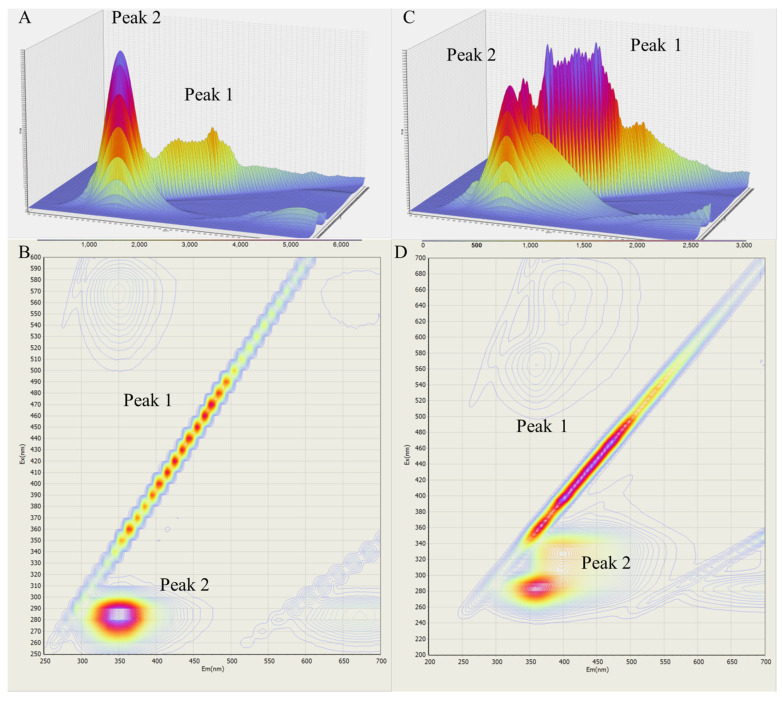
Three-dimensional fluorescence spectra of BSA (5 μM) in the absence (**A**,**B**) and presence (**C**,**D**) of *trans*-resveratrol (30 μM) at 298 K and pH 7.4. Ex = 200–500 nm, Em = 200–500 nm.

The three-dimensional (3D) fluorescence spectroscopic method facilitates depiction of the changes in the microenvironments of proteins and protein–ligand complexes [14]. Figure 5 presents the 3D profile of BSA with and without Res. Peak 1 is the Rayleigh scattering peak, and peak 2 is the characteristic peak of BSA. The profile of BSA and the BSA–Res complex differ, indicating the interaction between Res and BSA. The fluorescence intensity of peak 1 decreased significantly after the addition of Res (30 μM), indicating the formation of the BSA–Res complex and the alterations in the microenvironment of Tyr and Trp residues.

### 3.5. CD Spectroscopy

**Figure 6:**
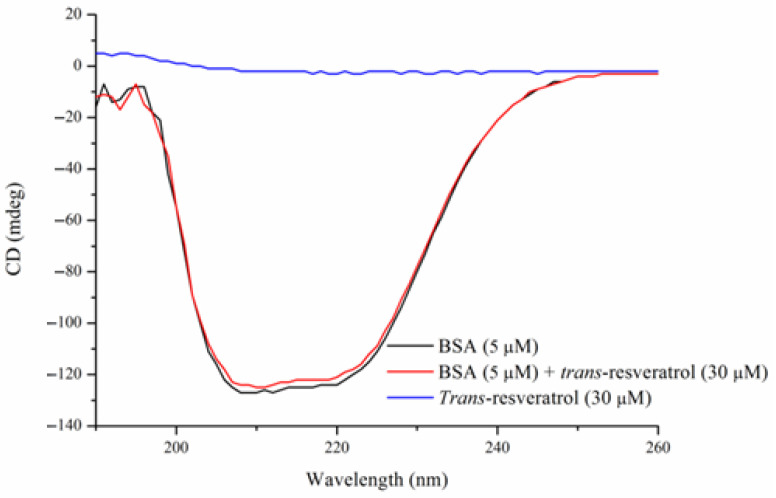
CD spectra of BSA (5 μM) and BSA–Res complex (BSA: 5 μM; *trans*-resveratrol: 30 μM). T = 298 K, pH = 7.4.

CD spectroscopy allows the secondary structures of proteins to be deciphered [15,18,25]. The spectra of BSA (5 μM), the BSA–Res complex (molecular ratio of 1:6), and Res (30 μM) were recorded, as shown in Figure 6. Two negative peaks at 208 (π-π) and 222 nm (n-π*) were detected in both the BSA and BSA–Res complex spectra, suggesting the α-helix structure of BSA. The α-helix content of BSA was 61.3%, near the result (62%) reported in the literature [26]. The α-helix content of the BSA–Res complex was 59.8%, slightly lower than that of BSA (61.3%), demonstrating that the interaction of Res and BSA caused a decrease in the α-helix content of BSA. Qi et al. found that the BSA–lutein dipalmitate complex decreased the α-helix content of BSA from 57.67% to 51% [15]. In another study conducted by Hussain et al., the α-helix content of BSA and BSA–β-resorcylic acid complex was 62.5% and 57.3%, respectively [14]. However, when β-resveratrol was bound to β-lactoglobulin, the secondary structure of β-lactoglobulin exhibited no remarkable change [23].

### 3.6. Binding Site of BSA

**Figure 7:**
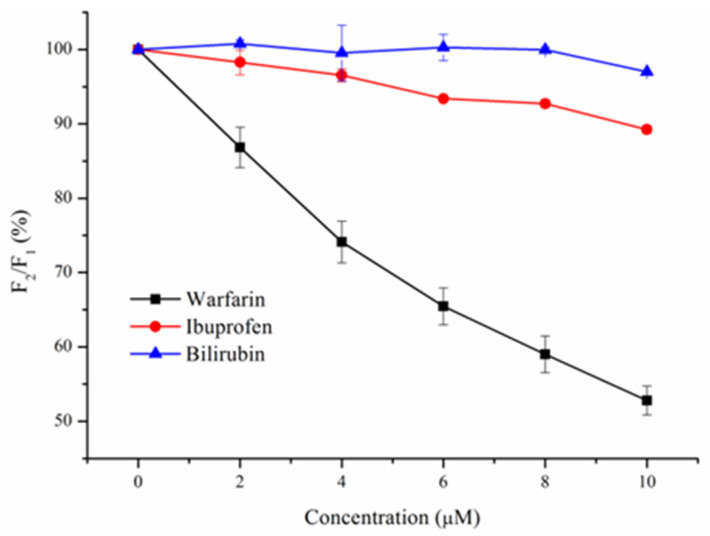
Effects of warfarin, ibuprofen, and bilirubin on the fluorescence of the BSA–Res complex.

According to previous studies, BSA has three main binding sites: I, II, and III, located in subdomains IIA, IIIA, and IB of BSA, respectively [12,16]. Known site marker fluorescence probes, such as warfarin, ibuprofen, and bilirubin, can be used for the identification of binding sites. Concretely, warfarin binds to site I (IIA), ibuprofen binds to site II (IIIA), and bilirubin binds to site III (IB). The F_2_/F_1_ value is usually used to determine the binding site. In this study, the F_2_/F_1_ values of warfarin, ibuprofen, and bilirubin were found to be 52.8%, 89.2%, and 96.9%, respectively, demonstrating that Res is bound to site I (Figure 7). In a similar study conducted by Qi et al., lutein dipalmitate was also found to bind to site I of BSA [15]. Hussain et al. performed a competitive binding study of the BSA–β-resorcylic acid complex using warfarin, ibuprofen, and bilirubin, and showed that β-resorcylic acid bound to site II of BSA [14].

### 3.7. Molecular Dynamic Simulation

The MD simulation was carried out to decipher the dynamic behaviors of the BSA–Res complex. The root mean square deviation (RMSD), root mean square fluctuation (RMSF), radius of gyration (R_g_), solvent accessible surface area (SASA), binding free energy, total energy, and decomposition energy were computed.

**Figure 8:**
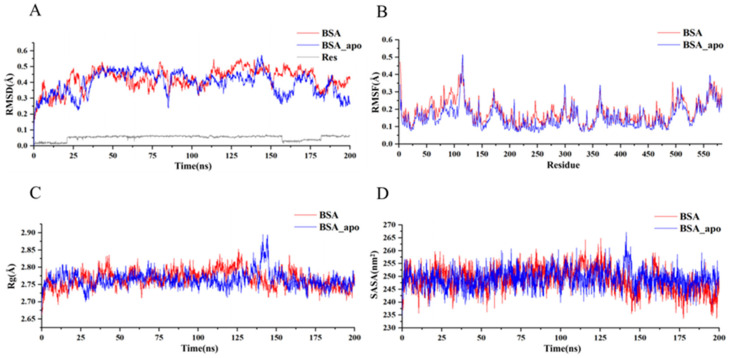
RMSD (**A**), RMSF (**B**), R_g_ (**C**), and SASA (**D**) of BSA in the absence and presence of *trans*-resveratrol.

The RMSD is used to assess the astringency and stability of protein molecules [14]. As shown in Figure 8A, the RMSD profiles of BSA and the BSA–Res complex showed a slight fluctuation during the entire simulation (200 ns). Furthermore, the BSA–Res complex had a higher RMSD value (0.420 Å) than BSA (0.394 Å). These results demonstrate that the conformations of BSA and the BSA–Res complex were stable, and that Res changed the conformation of BSA.

RMSF reflects the rigidity and flexibility of protein molecules, especially in terms of their amino acid residues [14]. The RMSF profile showed that amino acid residues of BSA and the BSA–Res complex exhibited tiny fluctuations in the simulation process (<0.52 Å). The results demonstrate that the conformations of BSA and the BSA–Res complex were stable and rigid during the interaction (Figure 8B).

R_g_ depicts the compactness of protein molecules. The difference in R_g_ values between BSA (2.764 Å) and the BSA–Res complex (2.766 Å) was lower than 0.28 Å in this study, indicating the slight conformational change in BSA after interacting with Res (Figure 8C).

SASA is one of the most important parameters for determining the stability of protein molecules [14]. Protein molecules with higher SASA values possess higher water solubility. In this study, the change in SASA was measured to confirm the stability of BSA and the BSA–Res complex. The SASA values of BSA and the BSA–Res complex exhibited mild fluctuations, with values less than 255 nm^2^, indicating their stable conformations and the slight alteration of BSA after its interaction with Res (Figure 8D).

**Figure 9:**
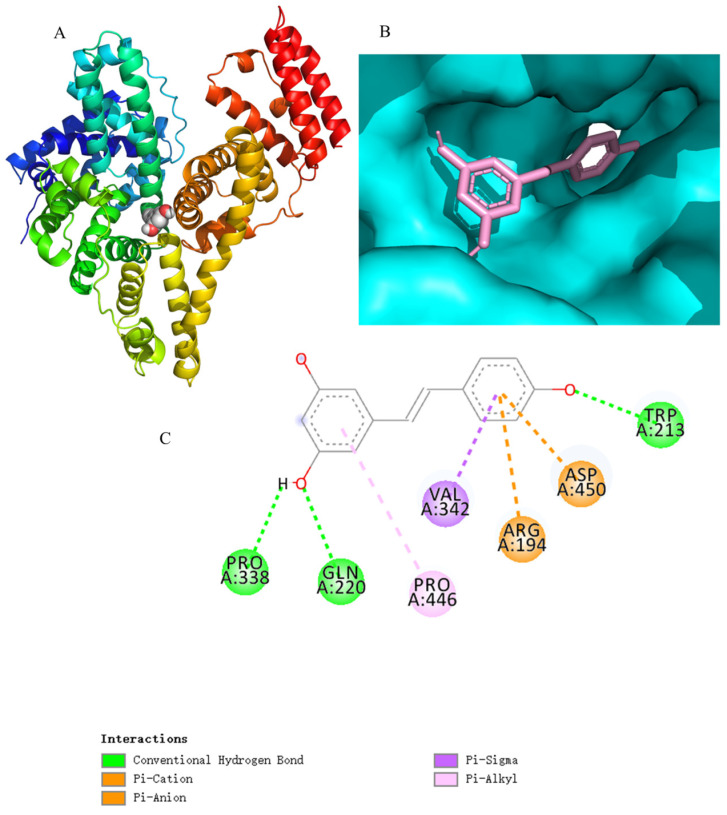
Combination model diagram after 200 ns in the molecular dynamics simulation (**A**). The rainbow color ribbon represents the BSA–Res complex, the yellow ligand represents Res, and the gray ribbon represents BSA only. Interactions between Res and BSA (**B**). The dashed blue lines represent hydrogen bonds, and the green lines represent π-π or π–alkyl interactions. Two-dimensional representation of the conformation of the BSA–Res complex (**C**).

The binding mode and site of Res in BSA were computed using the Gromacs software (2020.3). The results illustrated that Res bound to site I of subdomain IIA in BSA (Figure 9A,B), and the binding free energy was −19.31 kcal/mol (Table 2). These observations are in line with the results from the competitive binding experiment using warfarin, ibuprofen, and bilirubin as probes. According to Figure 9C, Res linked to BSA through Agr194, Leu197, Ser201, and Trp213 via hydrogen bonds and π-π or π–alkyl interactions. Besides MD and molecular binding simulation, density functional theory calculations—another in silico technology—are also a powerful tool for investigating the interactions between low-molecular-weight compounds (e.g., ligands) and macro-compounds. For example, Hao et al. have explored the effects of ultrasound treatment on starch–theanine–EGCG complexes based on multi-scale structure and prediction of the interaction via density functional theory calculations [27].

**Figure 10:**
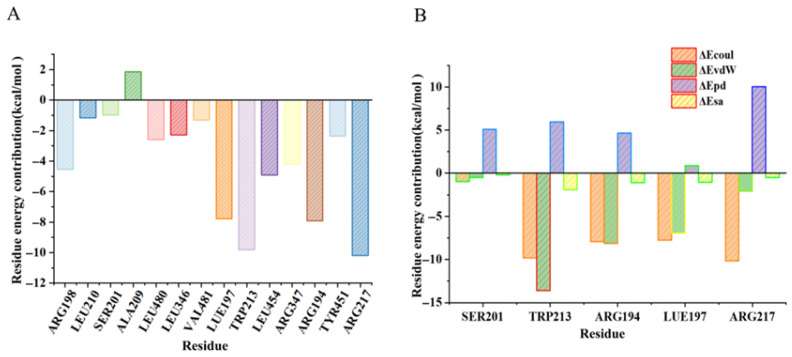
Per-residue total energy of BSA amino acids around bound Res (**A**). Per-residue decomposition energy of key amino acids in BSA around bound Res (**B**).

To further clarify the interaction of Res with BSA, the per-residue total energy and decomposition energy of BSA amino acids around the bound Res were also evaluated. As shown in Figure 10A, Arg217 and Trp213 exhibited the lowest total energy, with values less than −10 kcal/mol, while the values for Leu197 and Arg194 were around −8 kcal/mol. This observation is in agreement with the result shown in Figure 9C. Thus, the five main amino acid residues of BSA were computationally found to bind to Res. Coul energy and van der Waals energy were the main binding energies for Trp213, Arg214, and Leu197, while Coul energy and polar solvation energy were the binding energies for Arg217. Polar solvation energy was the most relevant binding energy for Ser201 (Figure 10B).

## 4. Conclusions

In conclusion, the interaction between BSA and Res was investigated through an integrated approach combining spectroscopic techniques and MD simulations. The binding process was determined to be spontaneous and characterized by a static quenching mechanism. Spectroscopic analyses revealed that Res binding induced conformational changes and microenvironmental alterations in BSA, with specific binding occurring at site I. The experimental findings were further validated and complemented through MD simulations, which provided molecular-level insights into the interaction dynamics. These results demonstrated the potential of BSA as an effective delivery system for Res. However, the in vitro or in vivo release kinetics of the BSA–Res complex and the binding ability of BSA with resveratrol analogs were not investigated. In the future, the release profile of resveratrol from BSA under physiological conditions, the binding of resveratrol analogs with BSA, and the delivery potential of such complexes, and multi-model comparisons should be investigated.

## Figures and Tables

**Table 1:** Quenching parameters of the BSA–Res complex at 298, 308, and 318 K.

T (K)	K_SV_ (×10^4^ M^−1^)	K_q_ (×10^12^ M^−1^ s^−1^)	R^2^ (Correlation Coefficient)
298	5.01 (±0.09)	5.01 (±0.09)	0.994
308	4.57 (±0.07)	4.57 (±0.07)	0.992
318	3.99 (±0.03)	3.99 (±0.03)	0.998

**Table 2:** Binding free energy of the BSA–Res complex (kcal/mol).

Complex	van der Waals Energy	Coul Energy	Polar Solvation Energy	SASA Energy	ΔG
BSA–Res	−133.52	−21.64	−82.07	−20.69	−19.39

## Data Availability

The original contributions presented in the study are included in the article/Supplementary Material, further inquiries can be directed to the corresponding author.

## Supplementary Materials

The following supporting information can be downloaded at: https://www.mdpi.com/article/10.3390/foods14142536/s1, Figure S1: The Stern–Volmer plot of F0/F versus the concentration of *trans*-resveratrol at 298, 308, and 318 K with pH = 7.4.
