# Supplementary material for: Interaction Between Bovine Serum Albumin and Trans-Resveratrol: Multispectroscopic Approaches and Molecular Dynamics Simulation
Source: Foods. 2025 Jul 20;14(14):2536. doi: 10.3390/foods14142536 (PMC12294381; doi:10.3390/foods14142536)
Supplement: Supplementary file 1 [file foods-14-02536-s001.zip › foods-3763738-supplementary.pdf]

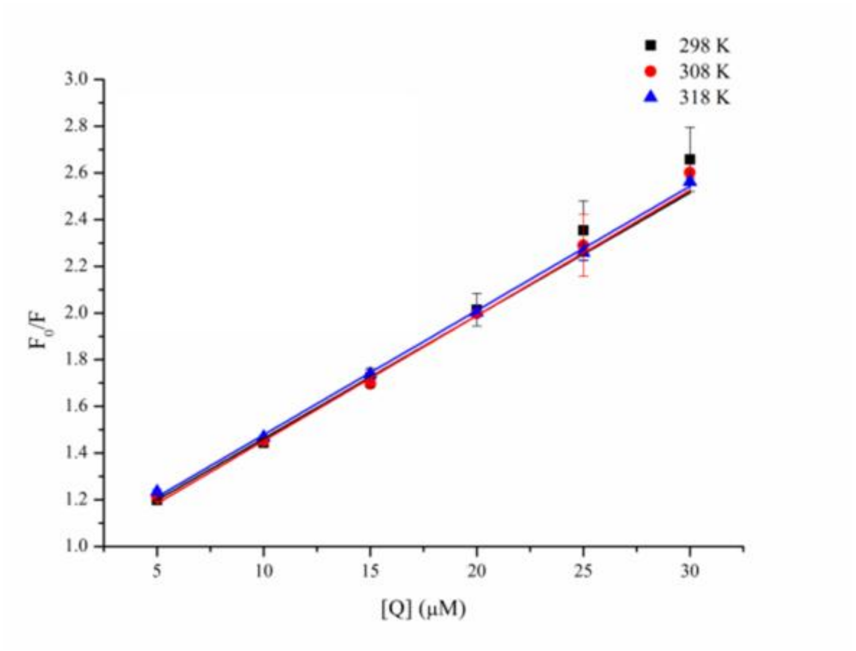

Figure S1 The Stern-Volmer plot of F_0/F versus the concentration of *trans*-resveratrol at 298, 308, and 318 K with pH = 7.4.
